# Influenza virus inoculum volume is critical to elucidate age‐dependent mortality in mice

**DOI:** 10.1111/acel.12893

**Published:** 2019-01-11

**Authors:** Candice A. Smith, Upasana Kulkarni, Judy Chen, Daniel R. Goldstein

**Affiliations:** ^1^ Department of Internal Medicine University of Michigan Ann Arbor Michigan; ^2^ Department of Microbiology and Immunology University of Michigan Ann Arbor Michigan; ^3^ Institute of Gerontology University of Michigan Ann Arbor Michigan

## Abstract

The elderly exhibit increased mortality to influenza viral infection for unclear reasons. Mice are frequently used to model how aging impacts disease. Several studies have shown that aged mice exhibit an increased mortality to influenza virus, but two recent studies demonstrated the opposite. These two studies administered the virus intranasally in 20 µL, whereas the other studies used a viral inoculum in at least 30 µL. To determine whether the volume of the inoculum could explain the conflicting reports, we infected young and aged mice via intranasal instillation of 40 µL or 20 µL containing 1 x 10^4^ plaque‐forming units (PFU) of H1N1 influenza virus. We found that intranasal administration of 40 µL but not 20 µL of inoculum resulted in age‐dependent mortality in mice. Compared to aged mice infected with 40 µL inoculum, those infected with 20 µL inoculum showed reduced levels of live virus and IFN‐β in the lung 3 days postinfection. Furthermore, aged mice administered 40 µL of Evans blue intranasally displayed increased dye retention in their bronchoalveolar lavage fluid compared to those administered 20 µL of Evans blue. Our data demonstrate that the inoculating volume of virus is critical for adequate delivery of influenza virus to the lung and thus for efficient infection of aged mice. These findings shed light on discrepant results in the literature regarding aged mice and influenza infection, and establish that mice can be used to examine how aging impacts the response to this biomedically important infection.

## INTRODUCTION, RESULTS, AND DISCUSSION

People over age 65 account for 90% of influenza‐related deaths (Pebody et al., 2010). Mice have been employed in hundreds of studies to examine how aging impacts the immune system (reviewed in Haynes & Swain, 2006; Nikolich‐Žugich, 2018) and in aging research in general (reviewed in Vanhooren & Libert, 2013). Discerning the mechanisms by which aging compromises the immune response to influenza in mice, and translating the findings to humans might inform on the development of novel therapies to reduce the suffering from influenza infections in the elderly.

The utility of the mice to study how aging affects influenza viral lung infection is somewhat controversial. At least five studies of either C57BL/6 mice or BALB/c mice report that aged mice (16–24 months old) display enhanced morbidity or mortality during influenza A virus (IAV) infection compared to young mice (2–4 months old), similar to humans (Steeg, 2016; Stout‐Delgado, Vaughan, Shirali, Jaramillo, & Harrod, 2012; Toapanta & Ross, 2009; Wong et al., 2017; Zhao, Zhao, Legge, & Perlman, 2011). In contrast, two studies of aged C57BL/6 mice (16–30 months old) report that aged mice exhibit reduced mortality during IAV infection compared to young mice (2–4 months old; Lu et al., 2018; Pillai et al., 2016). Interestingly, one distinguishing feature of these two studies was the use of viral inoculum in 20 µL, whereas the studies showing that aged mice succumb faster to IAV infection employed a viral inoculum in at least 30 µL. Additionally, a prior report in young mice showed that inoculating doses of virus <35 µL led to reduced mortality to the same dose of IAV (Miller, Kok, & Li, 2013). Given these differences, we reasoned that the volume for the viral inoculum could explain the discrepancy between the studies of aged mice.

**Figure 1:**
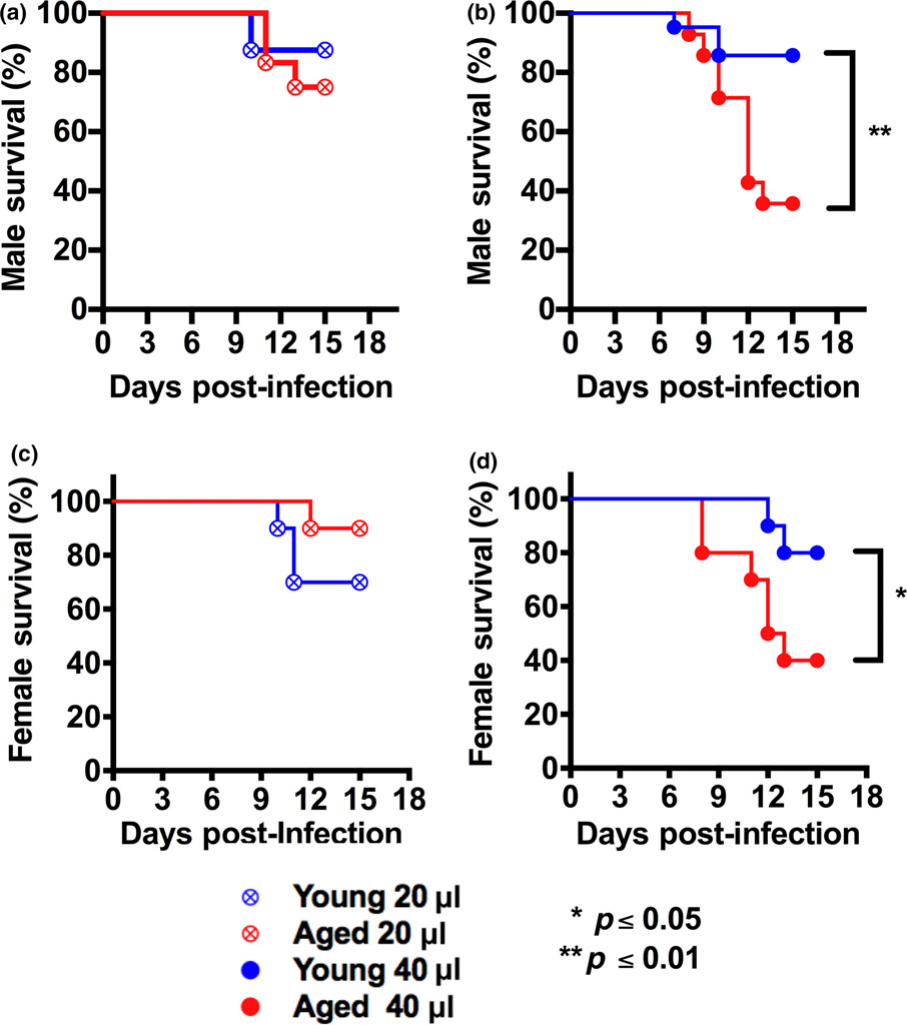
Volume of viral inoculum is critical for eliciting an age‐dependent mortality during IAV infection. Young (2–4 months of age) and aged (18 months of age) C57BL/6 male (a, b) or female (c, d) mice were infected i.n. with either 20 µL or 40 µL inoculum of virus containing 1 x 10^4^ PFU of PR8 strain IAV, and mortality was monitored. In both sexes, an age‐dependent increase in mortality was only noted with the 40 µL inoculum. *p < 0.05, **p < 0.01 (Gehan–Breslow–Wilcoxon test). *n* = 10–12/group (males), 10/group (females)

To test this hypothesis, we infected young (2–4 months) and aged (18 months) male and female C57BL/6 mice via intranasal (i.n.) administration of 1 x 10^4^ PFU of IAV (A/PR/8/34, H1N1) in either 20 µL or 40 µL of PBS and monitored survival. In mice infected using 20 µL inoculum, we did not observe age‐dependent alterations in survival in either sex (Figure 1a,c). In contrast, both sexes of mice infected using 40 µL showed a significant, age‐dependent decrease in survival (Figure 1b,d). As an alternative comparison, both sexes of aged mice infected with 40 µL showed significantly reduced survival relative to the same cohort infected with 20 µL (aged male *p* = 0.04; aged female *p* = 0.01, Gehan–Breslow–Wilcoxon test), whereas the volume of inoculum did not significantly alter the outcome for young mice (Figure 1). Thus, the inoculating volume influences the outcome of IAV infection in aged mice more than in younger mice. Furthermore, an inoculum of 40 µL effectively induced mortality in aged mice and revealed an age‐dependent mortality during IAV that was not observed with the 20 µL inoculum.

**Figure 2:**
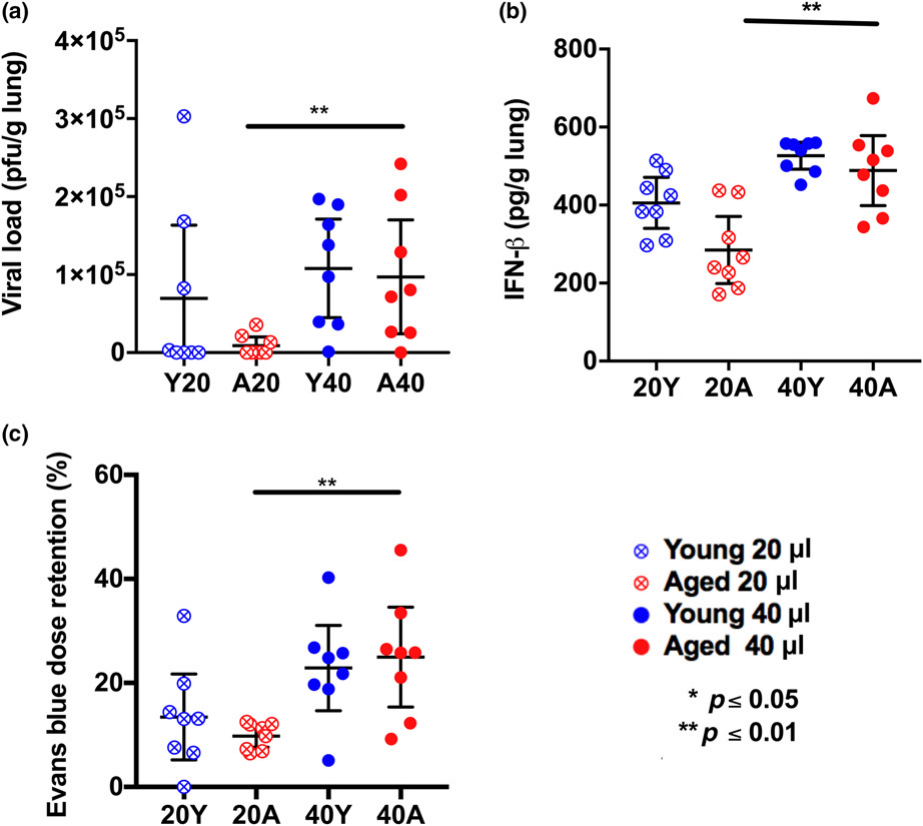
Increase in the volume of viral inoculum in aged mice via the i.n. route leads to higher viral load and increased IFN‐β levels. Increase in the inoculating volume of dye in noninfected aged mice leads to increased dye detection within bronchoalveolar lavage (BAL). Female young (2–4 months of age) and aged (18‐month old) C57BL/6 mice were infected i.n. with 1 x 104 PFU in either 20 µL or 40 µL, then live virus within lung tissue was measured 3 days postinfection by plaque assay (a) and concentration of IFN‐β measured in the lung lysate 3 days postinfection via ELISA (b). Young and aged noninfected female mice were administered 63 picomoles of Evans blue in either 20 µL or 40 µL, and dye was detected in BAL by colorimetry (c). *p < 0.05, **p < 0.01 (two‐way analyses corrected for multiple comparisons, see Supporting Information Appendix S1). Each data point represents a biological replicate, and error bars ±95% confidence interval

Next, we used viral plaque assay to measure the amount of live virus within the lungs. Again, we examined young and aged female mice infected with viral inoculum containing 1 x 10^4^ PFU of virus in either 20 µL or 40 µL. At 3 days postinfection, aged female mice administered viral inoculum in 40 µL displayed significantly more live virus in the lungs compared to aged female mice that received the 20 µL inoculum (Figure 2a). Furthermore, only 4 of 8 young female mice and 3 of 8 aged female mice administered viral inoculum in 20 µL displayed live virus in the lung, whereas all mice except one aged and young mouse administered viral inoculum in 40 µL exhibited live virus in the lung (Figure 2a). Together, these data imply a reduced efficacy of infection with 20 µL relative to 40 µL inoculum volumes, especially for aged mice, which would likely contribute to their increased survival upon infection with 20 µL vs 40 µL inoculum.

Type I interferons (IFN) are cytokines that are secreted early in response to viral infections including IAV infection (Asselin‐Paturel & Trinchieri, 2005). We measured the levels of IFN‐β, a key type I IFN cytokine, within the lung lysate of infected mice at day 3 postinfection. We found that the IFN‐β levels in lungs were almost two‐fold higher in aged mice infected using 40 µL compared to 20 µL viral inoculum (Figure 2b), even though both volumes contained 1 x 10^4^ PFU virus. These data further suggest that a 40 µL inoculum volume is more effective than a 20 µL inoculum volume for establishing a robust IAV infection, particularly in aged mice.

Prior work has shown that the volume of administration is critical for effective delivery of agents, including pathogens, to the lungs (Southam, Dolovich, O'Byrne, & Inman, 2002). The azo dye Evans blue has been used to measure the immediate efficacy of i.n. delivery in rodents (Visweswaraiah, Novotny, Hjemdahl‐Monsen, Bakaletz, & Thanavala, 2002). We used Evans blue to determine whether age and volume of administration impact the quantity of dye retained in noninfected mouse lungs. Noninfected aged and young mice were i.n. administered 63 picomoles Evans blue, in either 20 µL or 40 µL. Dye retention in the lungs was examined by harvesting bronchoalveolar lavage fluid (BAL) within 5 min and measuring Evans blue concentration by colorimetry. We found that aged female mice administered 40 µL Evans blue displayed a higher retention of dye in the lungs than those administered 20 µL (Figure 2c). Thus, using a 40 µL rather than 20 µL volume for i.n. administration leads to a significant increase in the delivery of dye and virus to the lungs in aged female mice (Figure 2a–c).

Overall, our study demonstrates that mice display an age‐dependent acceleration of mortality to IAV infection when the viral inoculum is in 40 µL. We found that using viral inoculum in a 20 µL volume will inadequately infect lung tissue, particularly for aged mice, confounding analyses. Although factors such as strain of virus, mouse strain, method of anesthesia, and route of administration of virus (i.n., or intratracheal) could also contribute to the variation across studies that use aged mice to study IAV infection, our study highlights the importance of the volume of viral inoculum. Upon administration of IAV in 40 µL, mice exhibit an age‐dependent increase in mortality similar to the clinical phenotype in humans. Therefore, our study confirms that mice are useful to model human aging and the outcomes to IAV infection.

## CONFLICT OF INTEREST

None.
