# Supplementary material for: Influenza virus inoculum volume is critical to elucidate age‐dependent mortality in mice
Source: Aging Cell. 2019 Jan 11;18(2):e12893. doi: 10.1111/acel.12893 (PMC6413738; doi:10.1111/acel.12893)
Supplement: Supplementary file 1 [file ACEL-18-e12893-s001.docx]

**Experimental Methods**

**Mice and *in vivo* viral infection**

C57BL/6 mice of 2-4 months and 18 months of age were obtained from the National Institute of Aging (NIA) rodent facility and kept in specific pathogen free biosafety level 2 facility. Mice received from NIA were the following weights: young females 22.6 ±1.8 g, aged females 26.5 ±2.0 g, young males 25.6 ±4.7 g, aged males 35.2 ±2.4 g. Mice were anesthetized with isoflurane and were then infected by i.n., with 20 μl or 40 μl PBS containing 1 X 104 PFU of influenza virus, A/PuertoRico/8/34 (H1N1) (PR8) from a commercial vendor (Advanced Biotechnologies) per our prior studies (JI 2017 *199*, 1060-1068). We did not observe any signs of respiratory distress in either young or aged mice, immediately following i.n administration of either 20 μl or 40 μl. Following infection, mice were monitored daily for changes in weight and mortality. Mice were euthanized when 30% of their original weight was lost, which was recorded as death in survival experiments. No animals were used in the study if they displayed other comorbidities prior to PR8 administration.

###### Viral load measurement

**Tissue Preparation:** Lung lysates from left lung lobe from mice were prepared in PBS using TissueLyser II (Qiagen). Following centrifugation at 400g for 10 min after which the supernatants were collected and stored at -80°C.

**Plaque assay:** Live lung viral titers were determined by plaque assay using Madin-Darby canine kidney (MDCK) cells (provided by Dr. Adam Lauring, University of Michigan). MDCK cells were cultured in DMEM medium at 37°C with 10% CO_2_ in 6 well plates (4 x 10^5^ cells/well) for 20-24 h until the cell monolayer reached 80-90% confluency. Lung lysate supernatants were thawed and 10-fold dilutions (10-1-10^-6^) were made with 0.1% BSA in 1x HBSS buffer (wash buffer). MDCK cells were washed twice with wash buffer followed by 1 h incubation with 400 μl of the 10-fold dilutions of lung lysate supernatants at 37°C with gentle shaking every 15 min. Cells were washed thrice with wash buffer to remove excess virus. The cells were then covered with 2 ml over-lay medium containing a 1:1 mixture of 2% agarose in sterile water and 2x DMEM medium containing 4 μg/ml acetylated trypsin (Sigma-Aldrich). Plates were incubated at 37°C for 72 h. Following 15 min incubation with 70% ethanol, the agarose was removed from the plates and the cells stained with 0.3% crystal violet for 5 min. The plates were washed and viral plaques were counted.

**Evans Blue Tracking Assay:** Evans blue powder was dissolved in 1x PBS and serial diluted create stocks of 0.3% and 0.15% Evans Blue. Healthy mice were anesthetized with isoflurane and administered 40 μL of 0.15% Evans Blue or 20 μL of 0.3% Evans Blue by i.n., route, equivalent to 63 picomoles Evans blue per dose. After 5 minutes, when the mice were fully awake and ambulatory, they were then euthanized by isoflurane overdose. Bronchoalveolar lavage was performed on the mouse lungs with 1.0 mL of 1x PBS twice and resulting bronchoalveolar lavage fluid (BALF) was stored in a centrifuge tubes. Two sets of calibration stocks were made by adding 40 μL of 0.15% Evans blue to 1.0 mL of PBS, and another where 20 μL of 0.3% Evans blue was added to 1.0 mL PBS. The stocks were each serial diluted to create calibration curves equivalent to the percentage of total dose administered to a single mouse. 100% of total dose would be equivalent to the [Evans Blue] in calibration stock. An 8-point calibration curve ranging from 0-100% total dose was created, and transferred to the plate in 100 μL aliquots in duplicate. The undiluted BALF from each treated mouse was transferred to the plate in 100 μL aliquots in duplicate. Concentration of Evans blue was measured by spectrophotometry at a wavelength of 620 nm. The resulting absorbance data was used to create calibration curves and to calculate the concentration, percentage of dose retained in lung, of Evans blue in the BALF.

**Quantification and statistical analysis:** Statistical analyses for survival were carried out using Gehan-Breslow-Wilcoxon test. For non-survival experiments, each unique animal sample was tested in duplicate and resulting values were averaged and resulting data point used for figures and further statistical analyses. Resulting data sets were displayed in figures as the mean ± 95% confidence interval. Comparisons of data from the Evans blue experiment and interferon-beta ELISA were carried out using 2-way Anova followed by the post-hoc Tukey test, after data was confirmed to have normal distribution in the following analyses: D’Agostino & Pearson normality test, Shapiro-Wilk normality test, and KS normality test. Non-parametric data was analyzed by the non-parametric 2-way Friedman test followed by post-hoc Conover test for multiple comparisons. GraphPad Prism software was used for survival and 2-way Anova analyses. The Friedman test and post-hoc Conover test were performed using R studio and code from Salvator S. Mangiafico (Mangiafico, S.S. 2016. Summary and Analysis of Extension Program Evaluation in R, version 1.15.0.). *P* values were considered significant if < 0.05.
